# Normal MRI features of the manica flexoria in horses and evaluation of the anatomic variability between forelimbs and hindlimbs

**DOI:** 10.1371/journal.pone.0327880

**Published:** 2025-07-21

**Authors:** Samantha Miles, Charles McCauley, Mariano Carossino, Fabio Del Piero, Chin-Chi Liu, Lorrie Gaschen

**Affiliations:** 1 Department of Veterinary Clinical Sciences, School of Veterinary Medicine, Louisiana State University, Baton Rouge, Louisiana, United States of America; 2 Department of Pathobiological Sciences and Louisiana Animal Disease Diagnostic Laboratory, School of Veterinary Medicine, Louisiana State University, Baton Rouge, Louisiana, United States of America; CONICET: Consejo Nacional de Investigaciones Cientificas y Tecnicas, ARGENTINA

## Abstract

Manica flexoria tears are increasingly recognized as a cause of lameness in horses resulting in a need for improved pre-operative diagnosis. Partial tears are difficult to pre-operatively diagnose and thus research for diagnostics of manica flexoria tears has been seen in increasing frequency over the past decade, though a deficit of information exists for the features of this structure on MRI, which is best suited for evaluation of soft tissues. The goal is to perform an observational study on the morphometry of the normal manica flexoria prospectively and describe it retrospectively on MRI. Inclusion criteria includes: MRI of the metacarpophalangeal/metatarsophalangeal regions in non-lame limbs without MRI tendon abnormalities identified, and the entire manica flexoria included on transverse proton density-weighted images. The manica flexoria on MRI was measured at the proximal and distal margins, halfway between (middle), and halfway between the middle and proximal margin (proximal fourth), with measurements including dorsal, lateral/medial and dorsolateral/dorsomedial thickness. Eighteen MRI studies fit the inclusion criteria: 6 forelimbs, 12 hindlimbs. The manica flexoria on gross dissection was thicker proximally in forelimbs, and thinner proximally in hindlimbs where it blends with overlying fascia. On proton density-weighted images, the manica flexoria was hyperintense to the superficial digital flexor tendon (12/18), isointense (3/18), or hyperintense proximally and isointense distally (3/18). The proximal fourth dorsal measurements were the thickest in both forelimbs and hindlimbs on MRI images compared to other measurement sites within the same limb. The forelimb medial aspect was thicker than the lateral aspect in the proximal fourth and middle (average 17.1% thicker, p = 0.0372, 22.7% p = 0.0183, respectively), and in the hindlimb the lateral aspect was thicker in these regions (average 50.4% thicker p = 0.0099 and 23.7% p = 0.0394, respectively). This study provides an anatomical and morphometric reference for future studies evaluating abnormalities of the manica flexoria on MRI.

## Introduction

The manica flexoria is a band of tendonous tissue that originates from the medial and lateral borders of the superficial digital flexor tendon and surrounds the deep digital flexor tendon. It provides a gliding surface for the deep digital flexor tendon proximal to the proximal sesamoid bones [1]. It also functions to direct the deep digital flexor tendon along the proximal scutum as it extends distally along the palmar aspect of the metacarpophalangeal joint and plantar aspect of the metatarsophalangeal joint [1,2]. It is attached to the digital flexor tendon sheath proximally and has a free distal border [1,2].

Manica flexoria tears are increasingly being recognized as a cause of lameness in horses and are reported to occur more commonly in the hindlimbs [2–4]. These commonly result in tendon sheath effusion leading to variable lameness exacerbated by flexion and poor performance. This can be difficult to investigate clinically due to the variability in response to intrathecal local anesthetic [2,5]. Therefore, imaging becomes important to further investigate a cause of the effusion. The degree of injury to the manica flexoria can be variable, ranging from partial, subtle tears to complete separation of the manica flexoria from the superficial digital flexor tendon [2,3,6,7]. Partial tears may be more difficult to pre-operatively diagnose and thus research for diagnostics of manica flexoria tears has been seen in increasing frequency over the past decade, with evaluation of radiographic contrast tenography, ultrasound and computed tomographic tenography [4,6,8]. This has resulted in more innovative ways of evaluating the manica flexori but does not allow for the specific evaluation of the tendon architecture, as seen with MRI [9,10]. Ultrasound is often utilized as a first line diagnostic in evaluation of the digital flexor tendon sheath and effusion of this region. However, the accuracy of ultrasound in the prediction of a manica flexoria tear is poor, especially when evaluating partial tears [3,11]. This has led to the investigation of additional techniques in which to evaluate the manica flexoria with ultrasound, such as dynamic flexion and extension in the non-weightbearing limb to help improve the identification of partial tears [6]. However, there is a gap in the utilization of an additional imaging modality which may be utilized for a definitive diagnosis.

Magnetic resonance imaging (MRI) has been utilized for identification of many tendon injuries of the distal limb in horses [9,10,12–14]. This modality is proven to be superior to CT and ultrasound for the evaluation of subtle tendon and ligament injuries in horses and has seen increasing utilization with the more widespread availability of both low-field and high-field MRI units. This has allowed for more specific localization of soft tissue injuries as well as a means of monitoring healing [10,14,15]. However, to the authors’ knowledge, there is no study describing the normal appearance of the manica flexoria on MRI, despite its preferred use in evaluation of other distal limb tendonous and ligamentous injuries in horses, and its superior evaluation of soft tissues. Our goal is to perform morphometry of the manica flexoria in non-lame limbs, or limbs of horses with lameness localized to the foot, retrospectively using MRI and to describe the MRI features of the normal manica flexoria in order to create reference values for comparison in affected horses. Our hypothesis is that the manica flexoria will be thicker proximally compared to distally, as previously reported [12], and there will be statistically significant differences between the forelimb and hindlimb measurements.

## Methods

This study has two parts: the first is an observational anatomic study involving dissection of two forelimbs and two hindlimbs from one sound control horse; the second part is a retrospective descriptive cross-sectional study.

### Gross examination techniques

Gross anatomic dissection of the limbs was performed by two authors. The manica flexoria was dissected in two forelimbs and two hindlimbs. The limbs were obtained from a 6 year old Thoroughbred gelding euthanized for reasons unrelated to orthopedic disease and determined to be free of visible and palpable pathology of the distal limb and tendons by one author. Due to availability of specimens at the authors’ institution, and the scope of the study, only a single horse was utilized for the purpose of gross evaluation of the manica flexoria for anatomic correlation to the MRI images. Following evaluation of the morphology of the manica flexoria in situ, the intact manica flexoria was submitted for histology. Three histologic sections were acquired per limb and submitted for evaluation, including the proximal margin, middle and distal portions (level A, C and D in this study).

### Image evaluation

**Fig 1:**
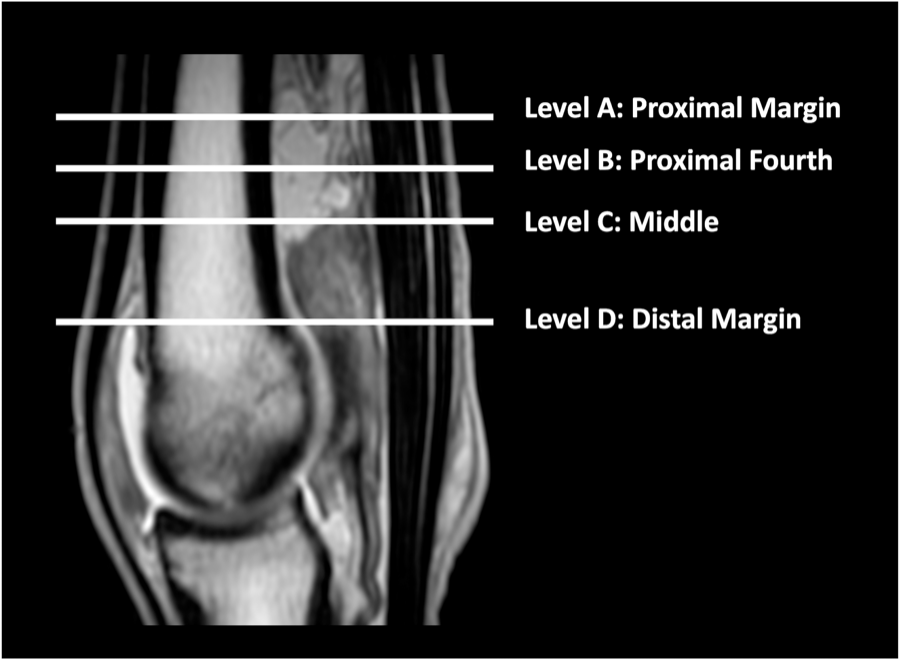
Measurement levels of the manica flexoria. Sagittal proton density weighted image of the metacarpophalangeal region in a horse with the levels used for measurement on transverse images depicted. Level A corresponds to the proximal margin; level B corresponds to the proximal fourth; level C corresponds to the middle manica flexoria (halfway between the proximal and distal margins); and level D corresponds to the distal margin.

**Fig 2:**
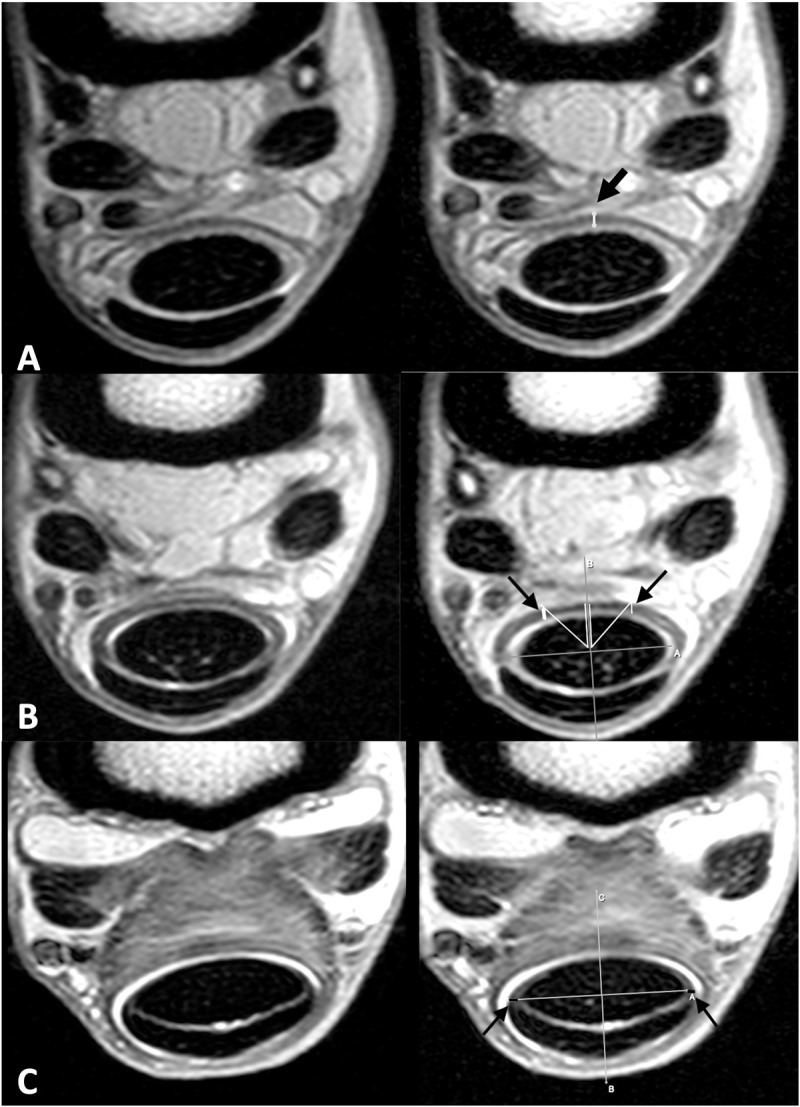
Measurements of the manica flexoria. MRI images of the normal manica flexoria of a forelimb at level A (A), level B (B) and level D (C) on the left with measurements used for this study on the right. Level C measurements are to the same as level B measurements. A) The proximal margin (level A) is measured from dorsal to palmar and recorded. This measurement was recorded at levels A, B and C. B) Level B is measured halfway between the proximal margin and middle and similar measurements were obtained at the middle of the manica flexoria (level C). A line through the sagittal plane of the deep digital flexor tendon and a perpendicular line through the maximal width of the deep digital flexor tendon are used to define the middle of the DDFT to more accurately measure between horses. The angle tool was used to create 45-degree angles from dorsal in order to measure the thickness at this location, noted by the white lines and black arrows. C) The distal margin (level D) lateral and medial margins are measured shown by the black lines and black arrows. Note the absence of the manica visible dorsally for measurement at this location. This measurement was recorded at levels B, C and D. Medial is to the left in all images.

MRI studies of the metacarpophalangeal and metatarsophalangeal joints from 2009–2020 were retrospectively reviewed. The MRI studies used included horses that had lameness that localized to the metacarpophalangeal or metatarsophalangeal region. The non-lame limb is always imaged at the authors’ institution as a comparison and these non-lame comparison limbs were used for this study. Additionally, horses were included when lameness was localized to the foot and the metacarpophalangeal and/or metatarsophalangeal joint was also included in the MRI study or separately imaged for screening purposes, without lameness localized to this region. All MRI images were acquired using a 1.5T magnet (Echelon, Hitachi Medical Systems America, Twinsburg, OH, USA) under general anesthesia with the horses in lateral recumbency. A 1.5T unit was utilized due to availability at the authors’ institution, and a high-field unit was preferred for this baseline study of the appearance of the manica flexoria due to its higher resolution. All MRI images were reviewed using an image analysis workstation (Horos, Nimble Co LLC, Purview, Annapolis, MD, USA). MRI sequences had the following parameters for proton density sequences: TE 12, TR 2500–5000 ms. MRI sequences had the following parameters for T2 sequences: TE 120, TR 5000–7500 ms. The matrix size was 320 x 320 and the field of view recorded was 13 x 13 cm. Inclusion criteria required transverse proton density weighted images including the entire manica flexoria from proximal to distal and absence of any tendon pathology on MRI. The images were evaluated by one author (a third year radiology resident). Using transverse views, the approximate length of the manica flexoria was determined by identifying the slice including the proximal and distal margins and calculating the number of slices including the manica flexoria, then multiplying the number of slices by the slice thickness. The manica flexoria was then divided into four portions based on the derived length: proximal margin (level A), proximal fourth (level B), middle (halfway between proximal and distal margins, level C) and distal margin (level D; Fig 1). The middle manica flexoria site (level C) was determined as the halfway slice between the proximal and distal margins seen on transverse proton density images. When this resulted in an odd number, the more distal slice was selected for measurement. The proximal fourth measurement (level B) was determined as the halfway point between the proximal margin where the manica flexoria is first visible (level A) and the site of the middle manica flexoria (level C; Fig 1). The following descriptions and measurements were recorded by one author: intensity of the manica flexoria relative to the superficial digital flexor tendon, uniformity, number of slices in which the manica flexoria was visible, slice thickness, dorsal measurements of the proximal margin, proximal fourth and middle manica flexoria (levels A, B and C), lateral/medial measurements of the proximal fourth, middle, and distal margin (levels B, C and D), and dorsolateral and dorsomedial measurements (45 degree angle thickness measurements) at the proximal fourth and middle manica flexoria (levels B and C; Fig 2). A line through the sagittal plane of the deep digital flexor tendon and a perpendicular line through the maximal width of the deep digital flexor tendon were made to divide this structure into four portions for accuracy of measurements for the dorsal, lateral and medial measurements, and this was used for the measurements at all levels (Fig 2). The angle tool was used to determine the 45-degree angle from the mid-point of the deep digital flexor tendon created by the lines described above for measurement of the manica flexoria at a precise angle for the dorsomedial and dorsolateral measurements. Additional T2 MRI sequences, when available, were also evaluated for visibility of the manica flexoria as well as intensity and uniformity compared to the superficial digital flexor tendon.

### Statistical analysis

All data analyses were performed by a Master of Applied Statistics and statistician using commercial software (JMP Pro 15.2.1, SAS Institute., Cary, NC). Measured values were assessed with a mixed analysis of variance (ANOVA) model with location of the manica flexoria, limb (forelimb vs hindlimb), and their measurements for each animal as the random effect. Assumptions of these models (linearity, normality of residuals, and homoscedasticity of residuals) and influential data points were assessed by examining standardized residual and quantile plots. When a fixed effect was detected, pairwise post-hoc LSD comparisons were performed for the effect. All significance was set at p < 0.05.

## Results

### Gross examination

**Fig 3:**
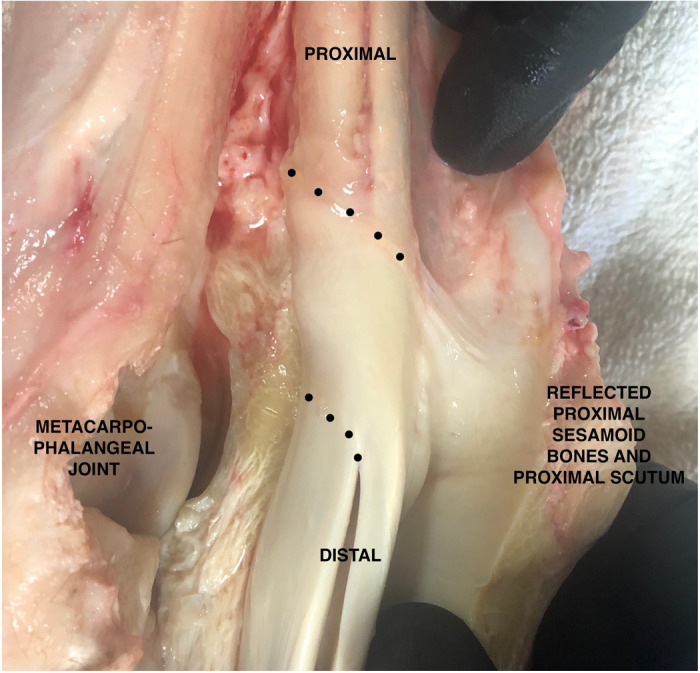
Gross anatomy of the manica flexoria margins. The forelimb manica flexoria dissected with the proximal sesamoid bones and proximal scutum dissected and reflected in a palmar direction, with dotted lines outlining the proximal and distal margins of the manica flexoria.

The manica flexoria is a thin structure extending from the lateral and medial margins of the superficial digital flexor tendon and surrounding the deep digital flexor tendons proximal to the proximal scutum and proximal sesamoid bones in both forelimbs and hindlimbs. The proximal margin is intimately associated with the loose connective tissue overlying the deep digital flexor tendon. The proximal margin of the manica flexoria is convex with sloping lateral and medial margins. This results in the lateral and medial margins at the junction of the manica flexoria with the superficial digital flexor tendon being distal to the proximal margin (Fig 3). This is opposite distally, with the dorsal portion of the distal margin of the manica flexoria being concave and terminating proximal relative to the lateral and medial margins, at their confluence with the superficial digital flexor tendon in both forelimbs and hindlimbs. The lateral and medial margins create a sharp angle as they merge into the superficial digital flexor tendon body (Fig 3).

**Fig 4:**
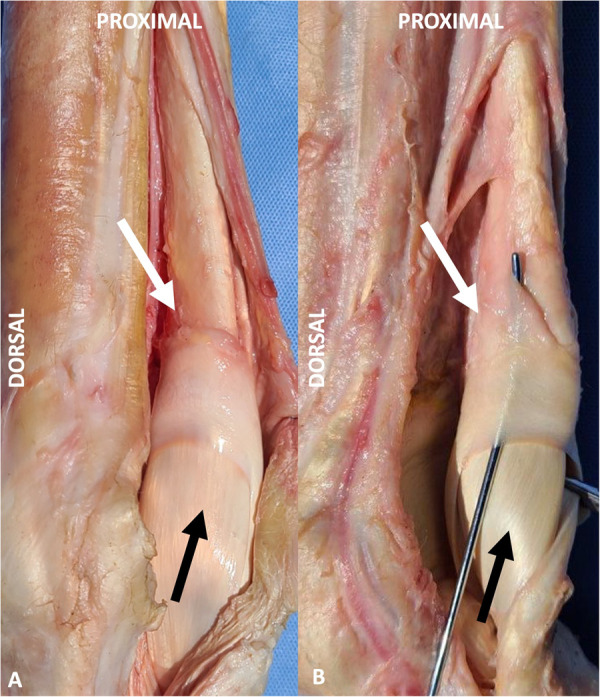
Gross anatomy of the manica flexoria and associated tendons. Dissected images of a forelimb (A) and hindlimb (B) manica flexoria (white arrow), with the flexor tendons reflected, depicting the dorsal margin of the deep digital flexor tendon and the adjacent manica flexoria. The deep digital flexor tendon is seen passing through this structure (black arrow). Note the convex proximal margin and the angled medial and lateral distal margins of the manica flexoria. In the hindlimb, note that the proximal margin of the manica flexoria is ill-defined and blends into the fascia overlying the deep digital flexor tendon.

In the forelimbs, the manica flexoria is subjectively thicker proximally than distally. In the hindlimbs, the proximal margin of the manica flexoria is subjectively thinner proximally relative to distally, blending with the overlying loose connective tissue of the dorsal aspect of the deep digital flexor tendon (Fig 4). Additionally, the manica flexoria is subjectively shorter in the hindlimb than in the forelimb.

### Histology

**Fig 5:**
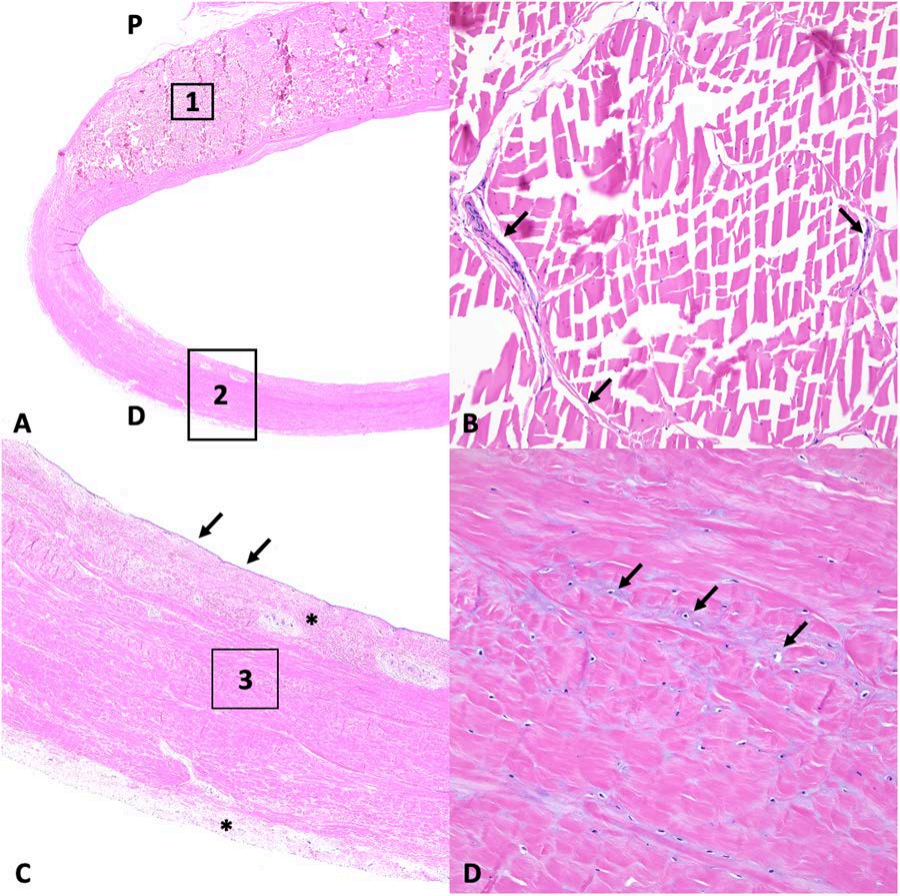
Histology of the manica flexoria. (A) Overview of the histological features of the manica flexoria and its connection to the superficial digital flexor tendon. The palmar/plantar aspect of the provided specimen (P) is delimited by the superficial digital flexor tendon, composed of bundles of dense collagen. The dorsal aspect of the specimen is composed of irregular, dense connective tissue. H&E, 10X total magnification. (B) The superficial digital flexor tendon is depicted as a magnified view of (A, 1). This represents the distal aspect of the superficial digital flexor tendon, which is composed of dense collagen fibers arranged in bundles and separated by thin, vascularized connective tissue septa (arrows). H&E, 200X total magnification. (C) The dorsal margin of the specimen represents the manica flexoria, depicted as a magnified view of (A, 2). The inner aspect is lined by flattened synoviocytes (arrows) with a layer of loose and vascularized connective tissue underlying the synoviocyte layer and along the superficial aspect (*). H&E, 50X total magnification. (D) Magnified view of (C, 3) along the dorsal aspect. Areas of fibrocartilaginous differentiation are noted (arrows). H&E, 200X total magnification.

The forelimb manica flexoria is composed of an outer layer of loose fibrous connective tissue extending from the dorsal aspect to the abaxial margins of the superficial digital flexor tendon and contains sporadic blood vessels. Underneath this outer layer of loose fibrous connective tissue at the dorsal aspect, the manica flexoria is composed of bundles of dense fibrous connective tissue with scattered fibrocytes characterized by condensed nuclear chromatin and separated by delicate septa of loose fibrous connective tissue with rare blood vessels (Fig 5). This layer is continuous with dense fibrous connective tissue along the palmar aspect but with no distinct separation of collagen bundles as seen in the dorsal aspect. Within this layer at the level of the palmar aspect, vaguely polygonal mesenchymal cells within lacunae suggesting fibrocartilaginous differentiation are present throughout the manica flexoria (Fig 5). This layer is poorly vascularized with very few blood vessels noted. Finally, the inner aspect is lined by a layer of thin loose fibrous connective tissue with scattered blood vessels and a layer of flattened synoviocytes (Fig 5). The hindlimb manica flexoria is similar in composition throughout, from proximal to distal. It is similar morphologically to the forelimb proximal fourth (level B), but having similar dense connective tissue centrally, bounded by dorsal and palmar regions of loose connective tissue.

### MRI evaluation

Eighteen MRI examinations of the metacarpophalangeal/metatarsophalangeal joints from 13 horses met the inclusion criteria, including 12 forelimbs (4 left, 8 right) and 6 hindlimbs (2 right, 4 left). Of these, there were 10 Thoroughbreds (7 forelimbs, 3 hindlimbs), 7 Quarter Horses (4 forelimbs, 3 hindlimbs), and one Selle Francais (1 forelimb). Ages ranged from 2–17 with a median age of 6. Slice thickness was 3 mm for 15/18 (83.3%) series and 3.5 mm for 3/18 (16.7%) with the latter being forelimbs. The manica flexoria was seen in a mean of 11 slices, with average length measurement based on slice thickness on MRI being 33.4 mm. There was no difference in average length or number of slices in which the manica flexoria was visible between forelimbs and hindlimbs.

**Fig 6:**
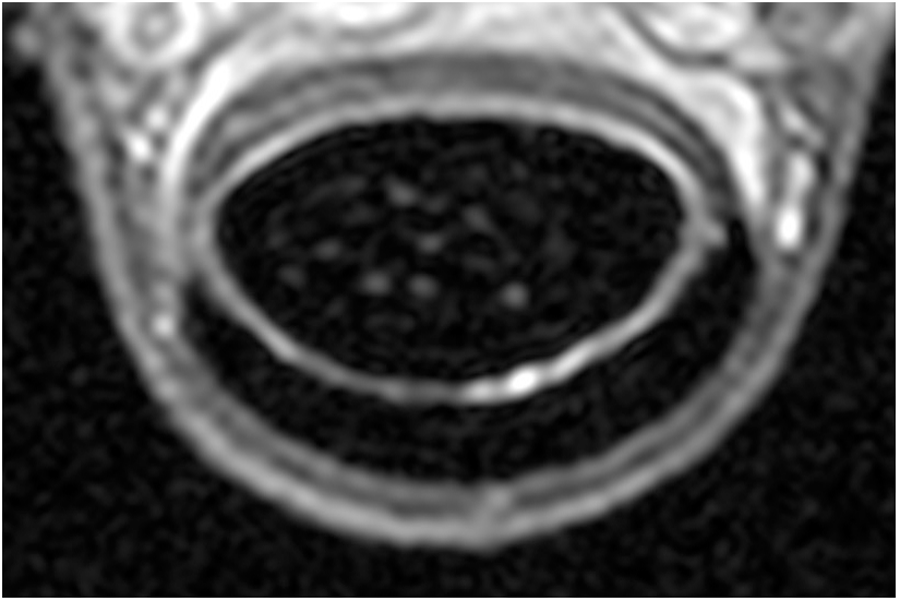
Transverse proton density weighted image of the manica flexoria. Transverse proton density weighted image of the forelimb of the horse at the level of the proximal fourth manica flexoria (level B) with the arrow pointing to a thin, hyperintense, smooth linear line parallel to the visible dorsal aspect of the manica flexoria. Medial is to the left.

The manica flexoria was homogeneously hyperintense in 12/18 (66.7%) PD sequences and homogeneously isointense in 3/18 (16.7%) PD sequences compared to the superficial digital flexor tendon. In 3/18 (16.7%) the manica flexoria was hyperintense proximally and isointense to the superficial digital flexor tendon from the middle aspect (level C) extending distally on PD sequences. In 16/18 horses (88.9%), a T2 weighted transverse image was available for review and in all these limbs (16/16, 100%) the manica flexoria was isointense to the superficial digital flexor tendon on T2 weighted images. In all proton density and T2 weighted sequences, a thin, well-defined, hyperintense, smooth linear structure was present in the middle and proximal fourth levels (B and C) of the manica flexoria parallel to the dorsal margin of the manica flexoria (Fig 6). In all limbs and sequences, the manica flexoria was smoothly marginated. On sagittal sequences, the manica flexoria was ill-defined or very thin resulting in variable visibility.

All means and standard deviations for measurements listed in Table 1. The thickest measurements were found at the proximal fourth (level B) dorsal measurement in both forelimbs and hindlimbs (p = 0.0002 and 0.0273, respectively), with a mean of 1.85 mm in the forelimbs and 2.01 mm in the hindlimbs. Comparing forelimbs to hindlimbs, the hindlimb dorsal measurement at the middle of the manica flexoria (level C) is greater than in the forelimbs at the same level (mean of 1.60 and 1.10, respectively; p = 0.0269). The remaining dorsal measurements were not statistically different between forelimbs and hindlimbs. In the forelimbs, the proximal fourth (level B) dorsal measurement was significantly thicker than the proximal margin dorsal measurement (level A), and the proximal margin dorsal measurement (level A) was thicker than the middle site (level C) dorsal measurement (mean of 1.85, 1.45, and 1.10 mm respectively, p = 0.0143 and <0.0001 respectively). In the hindlimbs, the proximal margin dorsal measurement (level A) was thinner than the proximal fourth dorsal measurement (level B, mean of 1.26 and 2.01 mm respectively, p = 0.0216). The proximal margin dorsal measurement (level A) was not significantly different from the middle site dorsal measurement (level C) in the hindlimbs (mean 1.26 and 1.60 mm respectively, p > 0.05).

**Table 1:** Measurement means and standard deviations.

Measurement locations in transverse plane	Location along manica flexoria (proximal to distal)		Forelimbs *Mean mm (SD)* *Range*	Hindlimbs *Mean, mm (SD)* *Range*
Dorsal	Proximal margin		1.45 (0.55)* 0.9-2.55	1.26 (0.53)^+^ 0.84-2.27
	Proximal fourth		1.85 (0.45)* 1.0-2.62	2.01 (0.29)^+^ 1.57-2.32
	Middle (halfway between proximal and distal margins)		1.10 (0.34)* 0.59-1.94	1.60 (0.38) 1.18-2.12
Lateral and Medial	Proximal fourth	Lateral	1.75 (0.41)^ 1.13-2.40	1.97 (0.34)^ 1.52-2.28
		Medial	2.05 (0.73)^ 1.2-3.52	1.31 (0.49)^ 0.84-2.18
	Middle (halfway between proximal and distal margins)	Lateral	1.76 (0.50)^ 1.1-2.5	2.14 (0.30)^ 1.75-2.64
		Medial	2.16 (0.68)^ 1.12-3.11	1.73 (0.53)^ 0.82-2.21
	Distal margin	Lateral	1.62 (0.49) 0.98-2.52	1.84 (0.38) 1.51-2.57
		Medial	1.80 (0.63) 1.07-3.19	1.59 (0.48) 0.78-2.20
Dorsolateral and Dorsomedial	Proximal fourth	Dorsolateral	1.83 (0.42) 1.29-2.46	1.77 (0.30) 1.43-2.31
		Dorsomedial	2.06 (0.61) 1.21-2.95	1.34 (0.46) 0.83-1.99
	Middle (halfway between proximal and distal margins)	Dorsolateral	1.42 (0.34) 1.03-1.98	1.79 (0.43) 1.13-2.16
		Dorsomedial	1.49 (0.35) 1.04-2.33	1.66 (0.47) 1.01-2.12

*These three values were significantly different from each other (p = 0.0002 from ANOVA p = 0.143 from post hoc: proximal fourth and margin, p < 0.0001 from post hoc: margin and middle, p = 0.0319 from post hoc: proximal fourth and middle).

+ These values were significantly different from each other (p = 0.0216 from post hoc).

^In all forelimb lateral and medial measurements, medial was greater than lateral at the proximal fourth and middle (p = 0.0372 and p = 0.0183 respectively); in all hindlimb lateral and medial measurements, lateral was greater than medial at the proximal fourth and middle (p = 0.0099 and p = 0.0394 respectively).

**Fig 7:**
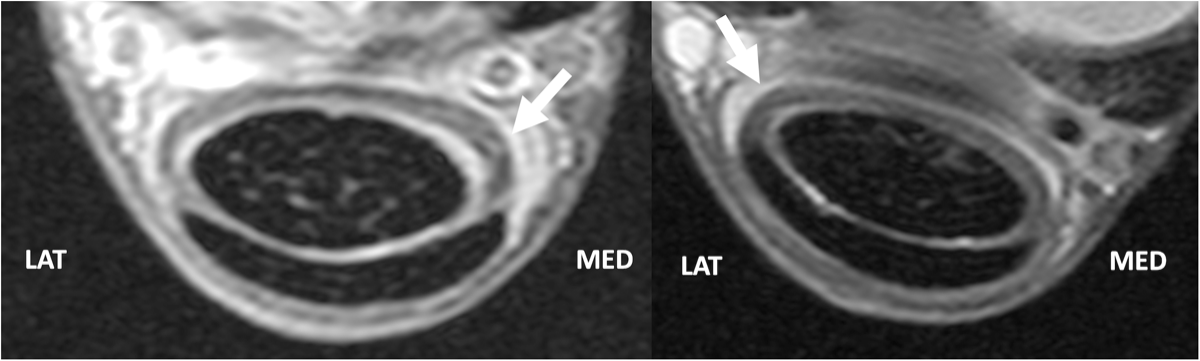
MRI images of the manica flexoria at level B comparing forelimb and hindlimb. Images from a forelimb (A) and hindlimb (B) at the proximal fourth (level B). Note that the medial aspect is thicker in the forelimb (A) and the lateral aspect is thicker in the hindlimb (B). Note the hyperintensity of the manica flexoria in both the forelimb and hindlimb compared to the superficial digital flexor tendon.

**Fig 8:**
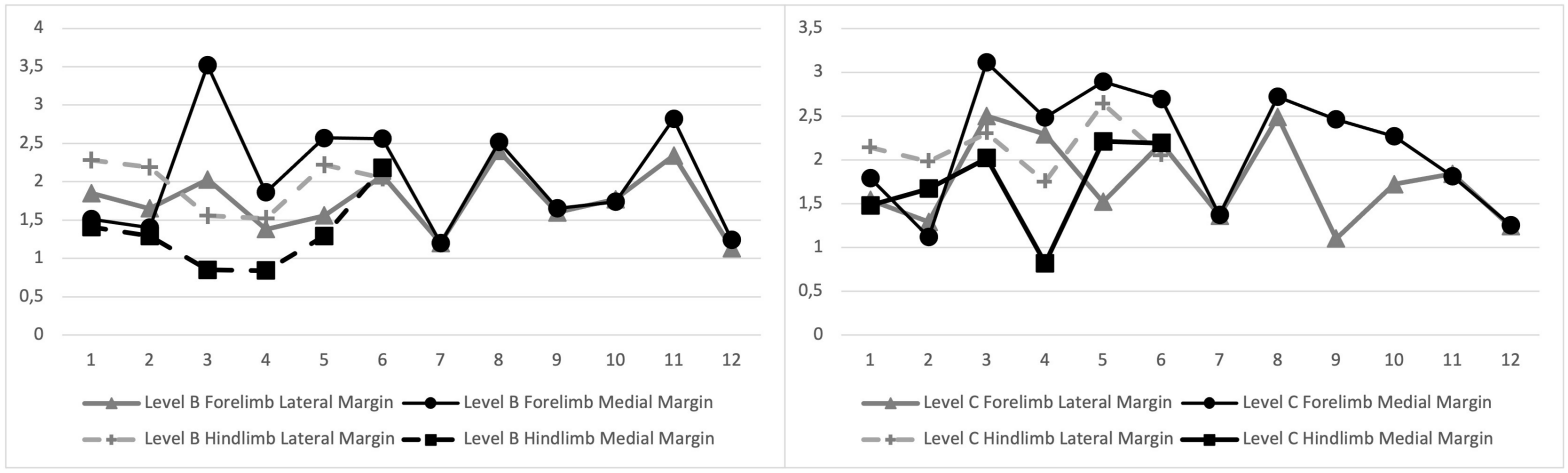
MRI images of the manica flexoria at levels B and C. (A) This graph depicts the medial and lateral margins of the manica flexoria at the proximal fourth (level B) of the manic flexoria. The dotted lines are utilized for hindlimbs and the solid lines are utilized for forelimbs. These lines depict that the medial margin is thicker in the forelimb relative to the lateral margin and the lateral margin of the hindlimb is thicker relatie to the medial margin. (B) This depicts the same as in (A) at the middle aspect of the manica flexoria (level C).

The forelimb manica flexoria medial aspect was thicker than the lateral aspect in the proximal fourth and middle, with a mean of 2.05 mm medially and 1.75 mm laterally in the proximal fourth, and 2.16 mm medially and 1.76 mm laterally in the middle (levels B and C, p = 0.0372 and p = 0.0183, respectively), Inthe hindlimb the lateral aspect was thicker in these regions, with a mean of 1.31 mm medially and 1.97 mm laterally in the proximal fourth, and 1.73 mm medially and 2.14 mm laterally in the middle (levels B and C, p = 0.0099 and p = 0.0394, respectively; Fig 7 and 8). There was no significant difference between the lateral and medial measurements of the distal margin in any limbs (level D). Comparing forelimb and hindlimb measurements, there was no significant difference at any level for medial or lateral margins between forelimbs and hindlimbs except at the proximal fourth medial measurement (level B) which was greater in the forelimb than the hindlimb (p = 0.0412).

The dorsomedial measurement was not significantly different between the proximal fourth and middle regions (levels B and C) for any hindlimbs. However, in the forelimbs, the proximal fourth location (level B) dorsomedial measurement was significantly greater than the middle (level C) dorsomedial measurement (mean 2.06 mm and 1.49 mm respectively, p = 0.0024). The dorsolateral margin of the proximal fourth (level B) was greater than the middle (level C) in the forelimbs (mean 1.83 mm and 1.42 mm respectively, p = 0.0081). When comparing dorsolateral to dorsomedial measurements, the forelimb dorsomedial measurement was greater than the dorsolateral measurement at the proximal fourth (level B, 2.06 mm and 1.83 mm respectively, p = 0.0181), with no significant difference between the dorsolateral and dorsomedial measurements in the hindlimbs. The middle (level C) dorsolateral and dorsomedial measurements had no difference in either forelimbs or hindlimbs.

## Discussion

To the authors’ knowledge, this study is the first description of the MRI features and morphometry of the manica flexoria and its correlation to the anatomy in vivo. Utilizing anatomic dissection of cadaver limbs allowed a thorough evaluation and description of the grossly normal manica flexoria and its subjective differences between forelimbs and hindlimbs. This allowed a greater understanding of the margins of the manica flexoria on transverse MRI images for evaluation in the second part of this study.

Significant differences in the thickness of the manica flexoria were found both within limbs and between forelimbs and hindlimbs, proving our hypothesis correct. The thickest portion of the dorsal margin of the manica flexoria was the proximal fourth (level B). This supports previous reports stating that the proximal aspect is thicker than the distal aspect [3,12,16]. In this study, in the proximal fourth and middle manica flexoria (levels B and C), the medial aspect was thicker in the forelimbs and the lateral aspect thicker in the hindlimbs, with no difference at the distal margins (level D). Manica flexoria tears have been reported both medially and laterally and it is unclear from previous studies if the distribution of these lesions correlates with the thinner aspects of the manica flexoria identified in this study [2,3,6]. In a study of 53 horses, a majority had injuries to the medial aspect of the hindlimb manica flexoria, however the study population was predominantly ponies and cob breeds. This study did not specify whether the forelimb distribution of lesions was different from the hindlimbs, and only reports the higher proportion of hindlimb and medial injuries [2]. In another smaller study, manica flexoria tears were more common laterally, but this study also did not differentiate between forelimb and hindlimb distribution [6]. However, this study population included Warmbloods as the predominant breed, with only two ponies. This may indicate that different breeds are predisposed to manica flexoria injuries in different regions and may account for the variability in the injury location prevalence between studies. Additionally, it is unclear if breed variation could also result in a varied appearance of the manica flexoria on MRI. It is possible that different breeds would have a different MRI appearance of the manica flexoria and the location of the thickest portion of the manica flexoria may also vary with different breeds. This may be especially true in breeds with considerable size variation from the cases in the current study, particularly ponies or draft horse breeds. The breeds presented in this study are representative of the population common to the authors’ institution. Unfortunately, the inclusion criteria of normal tendons on MRI limited the case numbers and, due to this, differences in breed could not be evaluated. Further studies with a larger number of a wide range of breeds, including ponies and cobs, would be needed to draw conclusions regarding whether the manica flexoria thickness is different between breeds and if the distribution of manica flexoria injuries differs between breeds and disciplines, and would require a large number of cases of each breed, at relatively similar ages for accurate comparison.

Manica flexoria tears are increasingly recognized as a cause of lameness and poor performance in horses. Research for diagnostics of manica flexoria tears has been seen in increasing frequency over the past decade, with evaluation of radiographic contrast tenography, ultrasound and computed tomography (CT). Radiographic contrast tenography was found to have high sensitivity for the diagnosis of manica flexoria tears but poor sensitivity for the commonly associated deep digital flexor tendon tears, limiting its usefulness as a sole imaging modality [4,17]. The manica flexoria has been described anatomically in multiple CT studies, both in vivo and ex vivo, with good success in identification of the normal manica flexoria, however pathology of this structure has not been fully described using CT [8,18,19]. Ultrasound is often utilized as a first line diagnostic in evaluation of the digital flexor tendon sheath. The accuracy of ultrasound in the prediction of a manica flexoria tear is poor, however it is very useful in the diagnosis of common concurrent lesions such as marginal tears of the SDFT or DDFT [3,6,11]. Additional techniques such as dynamic flexion and extension ultrasonographic evaluation of the manica flexoria has shown good initial results compared to standard ultrasound alone [6].

MRI has often been utilized for tendon and ligament injuries, allowing for the assessment of architectural changes [9,10,12,13,20], and is less invasive than radiographic and CT contrast tenography. This makes MRI an ideal modality, when available, for evaluation of the manica flexoria. Additionally, standing low-field MRI units are becoming increasingly popular and may allow for lesion diagnosis without the use of general anesthesia and its risks [21]. A previous study comparing standing low-field and high-field MRI of the metacarpophalangeal joint found that multiple, predominantly larger, anatomic structures, including the manica flexoria, were visualized with sharp margins on both high-field and low-field MRI [21]. However, this study used cadaver limbs in a standing MRI. It is unclear if the results of this study can be extrapolated to standing low-field MRI in live horses. It is possible that, given the decreased spatial resolution of low-field MRI relative to high-field MRI, as well as the added complication of motion the margins of the manica flexoria in a clinical case may be difficult to visualize and thus difficult to extrapolate these results. There is also limited published work on the appearance of manica flexoria tears on low-field MRI, being limited to work with cadaver limbs [9]. Further work would be needed to identify any differences between standing low-field and high-field MRI of the manica flexoria in vivo in both normal limbs and those with manica flexoria tears.

Proton density weighted transverse images were chosen for measurement in this study due to the improved visibility and confidence in identification of the manica flexoria compared to the T2-weighted transverse images and sagittal images. This is due to the improved spatial resolution of PD weighted imaging allowing more accurate evaluation of the margins of small structures relative to T2 weighted images, due to the high contrast nature of T2 images [22]. In all limbs, the manica flexoria could be reliably visualized from its proximal to distal margins, as this was an inclusion criterion. The manica flexoria has been reported to have low signal intensity [12]. However, no clarification was made as to whether there is variability between sequences or to what structures the manica flexoria intensity can be compared [12]. In this study, the manica flexoria was isointense to hyperintense compared to the superficial digital flexor tendon on PD-weighted images and isointense on T2-weighted images, thus allowing for objective comparison of intensity for future studies. The reason for the variability in intensity between limbs and horses is unclear. This may be due to differences in the type of collagen fibers. In a histological study of the manica flexoria, there was variation identified in the manica flexoria from dorsal to palmar/plantar, with variation in tenocyte and blood vessel morphology. For example, it was found that there is more fibrocartilaginous morphology of the tenocytes at the palmar/plantar aspects of the manica flexoria. The authors suggest this is due to the compressive loading of the manica flexoria at the palmar/plantar aspect adjacent to the deep digital flexor tendon during fetlock hyperextension [23]. This could also lead to variability in the intensity of the manica flexoria with age and training, if due to loading. However, complete evaluation of this is beyond the scope of this study and further studies among varied ages and breeds would be necessary for further investigation with further histological evaluation of the manica flexoria among different ages.

A limitation of MRI is limited spatial resolution that varies between sequences and changes with field strength. This can limit the measurement of small structures in which adequate spatial resolution is necessary for the accurate identification of margins, particularly the manica flexoria. This makes the measurement of this structure on all sequences difficult, and particularly on low-field MRI images, and may vary between sequences, particularly PD and T2 sequences, and measurements on other sequences should be interpreted cautiously [21,22].

The hyperintense line seen parallel to the dorsal margin in the center of the manica flexoria in all limbs most likely correlates to the central region of dense connective tissue seen on histology of our gross anatomic specimen. Hyperintensity of dense connective tissue on MRI has been reported in other structures, including the division of the lateral and medial lobes of the deep digital flexor tendon and the lateral branches of the suspensory ligament [12]. Connective tissue present between fibrils of tendons and at tendon margins have been reported to emit more signal [24]. The mesenchymal cells suggestive of a possible chondrocyte differentiation within the manica flexoria, identified in lacunae in the middle and distal segments of the forelimb, may be age-related change or represent a difference between forelimb and hindlimb manica flexoria morphology. Due to the nature of the study, anatomical dissection was performed in only one horse and this horse did not have correlative MRI images, thus a larger comparative histological and MRI based study may be useful for further investigation of the described findings. This would also allow further evaluation of age influences the morphology and MRI appearance of the manica flexoria. However, a previous study evaluating the histology of the manica flexoria found similar findings of blood vessels distributed within the dorsal and palmar/plantar aspects and chondrocyte-like tenocytes present within the manica flexoria of multiple horses, with no differentiation made between forelimbs and hindlimbs [23].

Hyperintensity in structures has also been reported due to magic angle artifact, caused by the angle of the evaluated structure relative to the magnetic field which can influence the intensity of structures asymmetrically and mimic pathology. However, this does not appear similar to the findings in this study, with magic angle artifact being a more diffuse hyperintensity than a thin linear line, as reported in this study. Additionally, magic angle artifact is most apparent on T1 weighted images, which were not used in this study [25,26].

Future studies would be needed to elucidate the effect of age on the MRI appearance of the manica flexoria. The age range of the patients in this study was wide, spanning from 2–17 years old. It has been previously stated that in young horses the manica flexoria is thicker on MRI [12]. However, the example given was anecdotal of a 6 month old foal with no range of ages to define young horses provided and no references provided [12]. Future studies including foals and horses of varying ages would be required to confirm that foals have a thicker manica flexoria and that it thins with age. The evaluation of a possible age variation in the thickness of the manica flexoria would also allow for future studies which would be necessary to elucidate the effects of training, the age of training initiation, and the age at the time of injury and their importance in relation to manica flexoria injuries. Additionally, it is unclear if the manica flexoria would also continue to thin with increasing age. Multiple studies report manica flexoria tears in horses of varied ages from as young as 6 years to 25 years of age, with approximate mean age of 13 years old [2,6]. If the manica flexoria continues to thin with age, it could explain the higher prevalence of manica flexoria tears in middle-aged horses. Further studies across a wide age range of the same breed would be necessary for reliable evaluation of these changes. The use of the specific regions for measurement utilized in this study could provide a basis for future studies to investigate if the manica flexoria thins uniformly or preferentially in a specific region with age, as well as comparison of hindlimb versus forelimb variability in the same horse.

Standard transverse sequences at the authors’ institution include PD-weighted, T2-weighted, and 3D T2*-weighted sequences. Unfortunately, given the length of time required for a 3D sequence, a small field of view is usually acquired and this is most often centered at the metacarpophalangeal or metatarsophalangeal joint, excluding a majority of the manica flexoria from this sequence. Therefore, the appearance of the manica flexoria on 3D T2* weighted sequences could not be evaluated. Additionally, T1-weighted sequences are not typically acquired at the authors’ institution, and further studies would be required to characterize the appearance of the manica flexoria on these sequences. It is unclear if the MRI appearance of the normal manica flexoria would be altered by surrounding pathology, such as digital tendon sheath effusion. The evaluation of pathologic changes was beyond the scope of this study and would require further investigation.

In conclusion, the normal manica flexoria is thicker proximally compared to distally in both forelimbs and hindlimbs, and is thicker medially in the forelimbs and laterally in the hindlimbs. A hyperintense line present parallel to the dorsal margin in the center of the manica flexoria in all limbs is most consistent with a band of dense connective tissue. Future investigations would need to be performed to elucidate whether manica flexoria injuries occur more frequently in the thinner medial aspect of the hindlimbs and lateral aspect of the forelimbs. This study provides an anatomical and morphometric reference for future studies evaluating abnormalities of the manica flexoria on MRI, which may improve pre-operative diagnosis of manica flexoria tears in the future, as well as assist in prognostication by identifying concurrent injuries.

## Supporting information

S1 File Raw data. (XLSX)
